# Case-Based fMRI Analysis after Cognitive Rehabilitation in MS: A Novel Approach

**DOI:** 10.3389/fneur.2015.00078

**Published:** 2015-04-08

**Authors:** Martina Hubacher, Ludwig Kappos, Katrin Weier, Markus Stöcklin, Klaus Opwis, Iris-Katharina Penner

**Affiliations:** ^1^Department of Cognitive Psychology and Methodology, University of Basel, Basel, Switzerland; ^2^Department of Neurology, University Hospital Basel, Basel, Switzerland

**Keywords:** working memory, cognitive training, rehabilitation, plasticity, multiple sclerosis, fMRI

## Abstract

**Background:**

Cognitive decline in multiple sclerosis (MS) negatively impacts patients’ everyday functioning and quality of life. Since symptomatic pharmacological treatment is not yet available alternative treatment strategies such as cognitive rehabilitation are of particular interest.

**Objectives:**

To analyse the ways in which MS patients respond to cognitive training, by combining behavioral and fMRI data in a case-based triangulation approach.

**Methods:**

Ten relapsing-remitting (RR) MS patients aged between 39 and 58 years and between 1 and 8 years post MS diagnosis were included. EDSS ranged from 1 to 3.5. Participants had normal to high intelligence levels. Six patients were assigned to the training group (TG) and four to the control group (CG) without intervention. The TG received a 4-week computerized working memory (WM) training, consisting of 16 training sessions of 45 min duration each. Before and after the training a neuropsychological examination and fMRI investigation by using an *N*-back task of different complexity was applied.

**Results:**

Patients in the TG responded differently to cognitive training. Four participants did not meet the triangulation criteria for being treatment responders. The two responders showed two distinct changes regarding activation patterns after training: (I) *decreased* brain activation associated with increased processing speed and (II) *increased* brain activation associated with higher processing speed and WM performance.

**Conclusion:**

The occurrence of different and opposed response patterns after the same training indicates a risk in applying classical group statistics. Different and especially opposed patterns within the same sample may distort results of classical statistical comparisons. Thus, underlying processes may not be discovered and lead to misinterpretation of results.

## Introduction

For decades, it has been known that patients with multiple sclerosis (MS) suffer from cognitive deficits. However, their importance for both the patients’ daily life and the overall health economy has been neglected for a long time. Meanwhile, they are regarded as a major element of the disease. Since symptomatic pharmacotherapy is not available, non-pharmacological approaches might further improve patients’ situation. In this context, cognitive rehabilitation has been studied with respect to its effectiveness. Several heterogeneous rehabilitation studies have been conducted, targeting either specific cognitive functions such as attention (1–4) or memory (5, 6) or applying a non-specific neuropsychological treatment. Primarily due to methodological heterogeneities, meta-analyses report negative results (7) or found only low evidence (8) for the effectiveness of cognitive rehabilitation approaches. Rosti-Otajarvi and Hämäläinen (9) report low but nevertheless positive evidence for cognitive training effects on working memory (WM) and other memory functions. However, clear evidence is missing, so far.

Studies using fMRI to monitor the effectiveness of cognitive treatment assume that behavioral improvement after cognitive training may be based on “adaptive” processes in the brain. Most studies report increased and more widespread activation in patients with MS after cognitive rehabilitation (1, 3, 10, 11). While patients receiving cognitive training show overall increased activation, untreated patients often show a decrease over time (12). However, a small trial, including only four participants with MS receiving cognitive training, reported increased activation in posterior regions but decreased activation in frontal areas of the brain (13) highlighting that brain adaptation is not only reflected by increased but also by decreased activation of task relevant areas.

In MS patients, aspects such as disease course, disease activity, cognitive status, fatigue, and depression can impact the responsiveness to cognitive interventions. This heterogeneity may result in different patterns of response to the same cognitive treatment. In trials with large samples, these influencing factors can statistically be controlled for, however, most rehabilitation studies only refer to small sample sizes. To address this problem, we propose a case-based approach to assess different patterns of response to cognitive training in heterogeneous and small samples. To underline the necessity of studying single patients more carefully, we present a case-series including six patients with early relapsing-remitting MS (RRMS) who received specific WM training during 4 weeks, and four control participants without intervention. The primary aim was to clarify whether (A) MS patients may show different brain activation responses to cognitive training and (B) how these changes in brain activation are finally related to individual cognitive performance. To answer these questions, a triangulation approach was applied.

## Materials and Methods

### Participants

Sixteen patients with CIS and early RRMS under interferon-beta-1b (Betaferon) therapy were recruited. Inclusion criteria were as follows: time since diagnosis <10 years, EDSS below 6.0, no relapses 3 months prior to the baseline visit. Participants were randomly assigned to the treatment group (TG; *N* = 9), or the control group (CG; *N* = 7), respectively. In the TG, two patients were excluded because they did not match the inclusion criteria and one was excluded because of an acute relapse during the intervention. From the CG, one patient quit the study because of personal reasons and two more participants were excluded because of relapses during the study.

The remaining 10 participants (TG = 6; CG = 4) were aged between 39 and 58 years and were between 1 and 8 years post MS diagnosis. Time since last relapse was shorter in the TG (0.25–4.4 years) than in the CG (2.7–6.4 years). EDSS ranged from 1 to 3.5. T2 lesion volume was between 0.24 and 8.53 ml. Participants had normal or high intelligence level. Baseline characteristics of participants are displayed in Table 1. The participants gave written informed consent to participate in the study, which was approved by the local Ethics Committee (Basel).

**Table 1:** **Baseline characteristics and possible factors for treatment response**.

Case	Gender	Age	Disease duration (years)	Number of relapses	Last relapse prior to study	EDSS	T2 lesion volume (ml)	General intelligence	Depressive symptoms	Cognitive fatigue	Motor fatigue
TGI	Male	47	2	6	9 months	3.5	0.27	96 PR	No	Severe	Severe
TG2	Female	44	1	2	3 months	1.0	5.93	50 PR	No	No	Moderate
TG3	Female	42	2	4	7 months	3.5	0.31	50 PR	No	Moderate	Severe
TG4	Male	42	5	2	4 years 5 months	2.0	0.24	99 PR	Mild	Moderate	Severe
TG5	Female	52	3	2	8 months	2.5	8.53	93 PR	No	Mild	Severe
TG6	Female	58	2	2	6 months	2.0	2.05	93 PR	No	Mild	Moderate
CGI	Male	46	8	4	6 years 5 months	1.0	1.43	99 PR	No	No	No
CG2	Female	42	3	3	3 years 2 months	2.5	1.38	73 PR	Moderate	Severe	Severe
CG3	Male	39	3	1	2 years 8 months	1.0	2.67	96 PR	No	No	No
CG4	Male	52	2	1	3 years 6 months	2.0	4.61	79 PR	No	Severe	Severe

*PR, percentile rank*.

### Study design

All participants underwent two baseline neuropsychological assessments within 2 weeks to assure a stable cognitive baseline status. During the second assessment, a baseline brain imaging (structural MRI and fMRI) was performed. All participants in the TG started their computerized cognitive training (BrainStim) within 1 week after the second baseline testing. They trained for 4 weeks, four times a week, for 45 min. Participants trained at home and were supervised once a week by a trained psychologist. Computerized training sessions were logged to monitor adherence to training. Participants in the CG received no intervention. Within 1 week after completion of the training, participants were retested for cognitive performance and a second MRI/fMRI was conducted.

To analyse the case series, a triangulation approach was applied as it is used in qualitative research (14). This methodological procedure combines quantitative and qualitative aspects (15). In order to measure a response to the treatment, detectable changes in more than one outcome parameter are taken into account. By applying this method to our case-series, response to treatment was defined by a combined change in brain activation on the one hand and cognitive functions (WM and/or processing speed) on the other. To overcome the problem of different scaling and to allow for direct comparisons between fMRI and cognitive outcomes, we intentionally avoided pre-defined cut-off values but focused on a qualitative description of changes by visual inspection.

### The cognitive training tool BrainStim

BrainStim (16) is a computerized training tool based on the WM model of Baddeley (17). It consists of three different modules targeting both, verbal and visual–spatial aspects of WM (18, 19). The first module trains spatial orientation. Participants have to memorize either a visually or verbally described route. This route has to be retraced on a virtual map afterwards. The number of crossings increases with higher levels of difficulty. A second module trains visual memory as well as the updating function of the central executive component. Participants have to remember the location of cards that have been turned over and back again. The task is to find pairs of cards with corresponding figures. With increasing levels of difficulty, the number of cards in one set is increases. During the third module, participants have to remember digits, presented in a limited period of time, and recall them after having performed an arithmetic distraction task. With each increase of the level of difficulty, more digits have to be recalled.

BrainStim is designed to ensure training not only based on repetition and practice but also on the development and consolidation of strategies. Therefore, the stimuli of the modules are presented randomly, where the order of the modules is changing in each session. The level of difficulty adapts automatically to the participants performance. After a pre-defined number of correct responses, the level of difficulty increases. Whenever the participant fails to solve a certain amount of tasks, the level of difficulty is decreases again.

### Cognitive assessment

At the first baseline visit, we collected demographical data and assessed premorbid intelligence [MWT (20)], fatigue [fatigue scale for motor and cognitive functions: FSMC (21)], and depressive symptoms [BDI-fast screen (22)]. Based on previous work (18) where BrainStim has proven its specific effect on WM and processing speed, we defined these functions as primary cognitive outcome measures. The *Corsi Block backwards* task was used for visual WM and the *Digit Span backwards* test for verbal WM [Wechsler memory scale-revised (23)]. The *symbol digit modalities* test (SDMT) was used to measure WM performance and processing speed (24). To receive a measure for processing speed that is not confounded with WM, we used the *alertness* tasks (tonic and phasic) from of the *test battery for attention performance* [TAP (25)]. Age corrected normative data was available for all cognitive tests. For WM (Corsi Block bw and Digit Span bw) as well as for alertness (tonic and phasic) percentile ranks <16 were regarded as a clinically meaningful cognitive deficit. SDMT scores were *z*-transformed according to Scherer et al. (26) and *z*-scores less than −1.68 were rated as clinically significant.

### fMRI paradigm

During fMRI, participants solved a *N*-back task with different WM loads [adapted from the TAP (25)]. Series of pseudo-randomized digits were continuously presented on a screen. Participants were asked to press a button as fast as possible whenever the target appeared. A target was a digit that was identical to the immediately preceding digit (1-back), the second to the last digit (2-back), or the third to the last digit (3-back). A block design was used for semi-randomized presentation of the *N*-back conditions and rest condition (fixation cross). One active block with a duration of 30 s consisted of 10 stimuli with 2 stimuli being targets. Each condition was presented four times during each session. Participants performed the paradigm two times with a break between the two sessions. In sum, each condition was presented during eight blocks. Reaction times for *N*-back tasks were logged, but due to technical problems this files were not available for all participants and time points and therefore excluded from further analysis. Immediately prior to the MRI, participants were familiarized with the *N*-back task outside the scanner to ensure comprehension.

### MRI data acquisition

The MR measurements were performed on a 3.0-T scanner (Magnetom VERIO, Siemens Healthcare, Erlangen, Germany) with a standard head coil. An anatomical image for registration purposes was acquired [sagittal T1-weighted 3D high resolution magnetization-prepared rapid gradient echo (MPRAGE) sequence: TR/TE/TI = 2000/3.37/1000 ms, 256 × 256 matrix, field of view (FoV) = 256 mm, providing an isotropic spatial resolution of 1 mm^3^]. For lesion masking, a T2-weighted fluid attenuated inversion recovery (T2-FLAIR) sequence was obtained (TR/TE/TI = 8000/77/2370 ms, 40 slices with slice thickness of 3 mm and FoV = 220 mm).

Echo-planar imaging (EPI) sequences were used for functional imaging (TR/TE = 2000/23 ms, 34 slices with a slice thickness of 3 mm, FoV = 256 mm, voxel size = 4 mm × 4 mm × 3 mm). Slices were positioned parallel the AC–PC line. For both runs with the paradigm, 262 volumes with a total scan time of 8.5 min were recorded. After excluding the 5 five dummy scans per run, 514 volumes remained for further analysis.

### MRI data management and analysis

Data were analysed using Statistical Parametric Mapping software package, SPM8 (http://www.fil.ion.ucl.ac.uk/spm). We identified T2 hyperintense white matter lesions with the lesion segmentation toolbox [LST (27)]. To choose the optimal initial threshold κ, lesion segmentation was run with different thresholds. Afterwards, two independent evaluators compared manually the resulting lesion maps with the original raw images. By this approach, an initial threshold of κ = 0.2 was chosen. Lesion masks were used for automatic lesion filling with intensities similar to the normal white matter voxels in T1-weighted images. We used these “lesion-free” T1-images for later registration steps. Further, the lesion-filled T1-images were segmented into gray matter, white matter, and CSF (“new segment”). Gray matter and white matter were fed to DARTEL to create a study-specific template (28).

fMRI data were realigned, unwarped, and co-registered with the T1-images. fMRI images were then normalized to MNI space with the corresponding DARTEL flow fields and a 8 mm Gaussian smoothing.

Since we were interested in changes between the two time points, all smoothed images were subject to a first-level analysis to define the model design and contrasts of interest. Movement parameters extracted from the realignment step were included as additional covariates in order to remove residual variance. Contrasts for changes between baseline MRI and the post-training MRI in each subject [*p* < 0.001, threshold: 10 voxels per cluster (29)] for all performance conditions (1-back, 2-back, and 3-back) were specified to identify activation increase and decrease between the times of measurement.

## Results

### fMRI assessment

**Figure 1:**
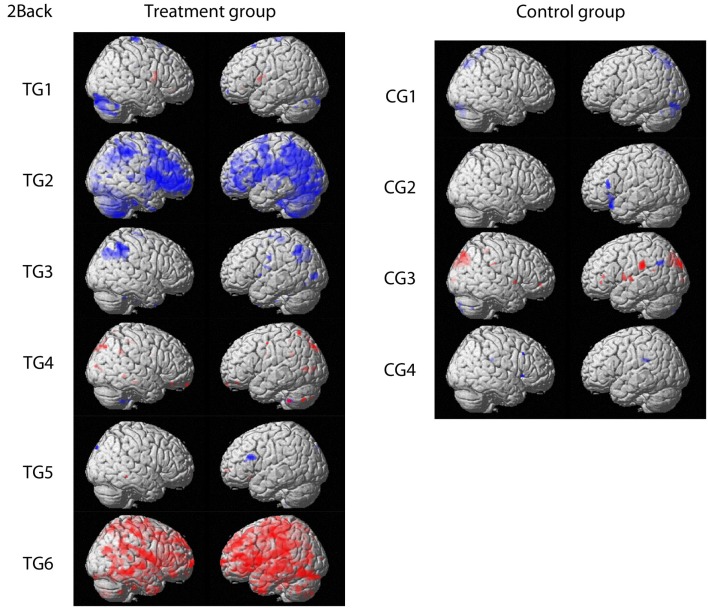
**Contrasts comparing baseline and post-training fMRI results for the treatment group and the control group, respectively**. Activation increase is marked in red whereas decreased activation over time is highlighted in blue (*p* < 0.001 uncorrected; threshold: 10 voxels per cluster). Figures are shown in radiological convention.

For fMRI outcomes, contrasts between baseline and post-training for 1-back, 2-back, and 3-back conditions for each participant were built individually. Patterns of response were comparable for the three conditions. Therefore, only contrasts from the 2-back condition are displayed in Figure 1 for clarity reasons (*p* < 0.001 uncorrected, threshold: 10 voxels per cluster). Four participants (TG1, TG3, TG4, TG5) receiving the training showed only minor changes in brain activation, which were comparable to changes observed in participants without training.

Two participants (TG2, TG6) showed changes in brain activation that exceeded changes observed in patients without training. One participant (TG2) showed decreased activation in primarily frontal and parietal regions. In TG6, the opposite pattern was observed. This participant showed increased brain activation spread across the whole brain except for the occipital lobe.

### Neuropsychological assessment

At baseline, no participants were impaired regarding tonic alertness and SDMT. Three participants (TG2, TG5, CG2) had reduced phasic alertness of whom one participant (CG2) showed reduced visual WM span (corsi block backward) in addition. One participant (TG1) showed reduced verbal WM performance (digit span backward). On a group level, by applying Mann–Whitney-*U* test performance on the digit span backward in the CG was higher than in the TG whereas no other baseline differences were detectable. (Note: Although this work is focused on qualitative single subject analyses the authors included this information revealed by group analyses on explicit request by one reviewer.)

For longitudinal comparisons, we used raw scores as displayed in Table 2. When comparing baseline and post-training results, none of the participants showed a consistent increase in all cognitive domains. One TG participant (TG3) showed solely an increase in the visual WM task. Participant TG1 performed faster during both alertness tasks. Two participants (TG2, TG4) showed faster reaction times in the alertness tasks and increased scores in the SDMT. TG5 had increased WM functions but no speed increase. TG6 performed better after the training in four of the five outcome measures. In the CG, two participants (CG1, CG3) showed increased verbal WM scores, one participant (CG2) had faster reaction times during the alertness task and higher visual WM scores. CG4 showed faster reaction times during the phasic alertness task. Three participants of the CG (CG1, CG3, CG4) had decreased reaction times during tonic alertness after 4 weeks. No participant of the CG showed changes in the SDMT task. On group level, there were no differences between the TG and the CG after the training. (Note: Although this work is focused on qualitative single subject analyses the authors included this information revealed by group analyses on explicit request by one reviewer.)

**Table 2:** **Raw scores of primary cognitive outcome measures for all participants at baseline and after the training**.

	Processing speed measures				Processing speed and WM (SDMT)		WM measures			
	Tonic alertness (TAP alertness A)		Phasic alertness (TAP alertness B)				Visual WM (corsi blocks bw)		Verbal WM (digit span bw)	

Case	Baseline	Post-training	Baseline	Post-training	Baseline	Post-training	Baseline	Post-training	Baseline	Post-training
TG1	266.5	242.0	251.5	217.0	50.5	54.0	9.5	10.0	5.0^a^	5.0
TG2	285.5	260.0	280.5^a^	264.0	65.0	75.0	9.0	9.0	6.0	5.0
TG3	293.0	293.0	247.5	248.0	56.5	59.0	7.5	9.0	6.0	7.0
TG4	242.0	227.0	233.5	204.0	45.5	61.0	10.0	10.0	7.0	8.0
TG5	276.5	271.0	312.0^a^	307.0	57.5	66.0	8.5	10.0	5.0	8.0
TG6	255.0	246.0	258.0	242.0	63.0	72.0	8.5	10.0	5.0	6.0
CG1	214.0	223.0	220.5	223.0	56.5	55.0	9.0	10.0	9.5	11.0
CG2	272.0	243.0	301.5^a^	235.0	56.5	59.0	7.0^a^	9.0	7.0	7.0
CG3	235.5	249.0	228.0	232.0	65.5	67.0	10.0	11.0	8.0	11.0
CG4	235.0	268.0	259.5	243.0	47.5	43.0	7.0	8.0	6.0	5.0

*Improvements from baseline to post-training are highlighted in gray*.

*Alertness (TAP) scores represent reaction times*.

*bw, backward; WM, working memory; TG, training group; CG, control group*.

*^a^Clinically meaningful baseline values (PR < 16 for alertness and WM tasks; *z* < −1.68 for the SDMT)*.

## Discussion

To identify possible effects of WM training on brain functionality and cognitive status, we presented six cases with RRMS receiving WM training during 4 weeks and four control cases without intervention. At a purely descriptive level, the key differentiators between TG and CG were SDMT and tonic alertness. Four out of six cases in the TG were able to increase their performance on the SDMT, whereas no participant in the control condition did so. Regarding tonic alertness, four of six participants in the TG showed higher performance after the training whereas three out of four participants in the CG showed even a performance decrease.

Regarding functional brain activation, four TG participants showed only minor changes in brain activation, which were comparable to changes observed in the CT. We therefore conclude that these minimal changes reflect a normal range of variation during a 4 weeks period and not a response to training.

Two TG participants met our triangulation criteria for being responders: both changes in brain activation and changes in WM or processing speed measures were observed. One participant showed a *decrease* in activation during the training period in frontal and parietal regions. The other responder showed an *increase* in brain activation in frontal and parietal regions as well as an additional increase in temporal regions.

The opposed response to treatment measured by fMRI might be reducible to different brain processes. Plasticity processes related to practice have been studied intensively in healthy individuals. Group analyses regarding short-term WM training (duration of training: 30–120 min in total) in healthy adults revealed decreased brain activation in frontal (dorsolateral, prefrontal, inferior frontal, precentral sulcus) and parietal regions (30–33), whereas more intense training led to mixed patterns of increases and decreases (34–37). In their review article, Kelly et al. (38) described four different patterns of change in brain activation due to practice: decrease, increase, redistribution, and reorganization. In a subsequent meta-analysis, Buschkuehl et al. (39) described the same patterns of response to WM training:

(1) Decrease in extent or strength of activation within one network that is associated with higher performance has often been reported after short-term training, mainly based on practice (30, 32). It is thought to be associated with a certain sharpening of response within the network where less neurons are firing in response to a task. This change might reflect more efficient information processing in the brain. The decrease of brain activation within the WM network in one of our responders might be related to this process. This change in activation was accompanied by an increase in processing speed on the behavioral level (Alertness and SDMT; for summary see Table 3). Thus, this increased processing speed can be regarded as the behavioral expression of more efficient information processing within the brain.
(2) A second pattern is referred to increased activation within one network (40). Here, increased intensity of activation is thought to be associated with a strengthening in response to a specific task, whereas increase in extent of the activated network reflects additional recruitment of cortical units. None of our participants showed a comparable change in brain activation.
(3) Combined increase and decrease within a network might occur in response to cognitive training (34, 41). This is referred to as redistribution of activation. The same cognitive process is used to solve the task, but due to practice and learning less attention control is needed and task specific processes are more involved. None of our participants showed a similar pattern of change in brain activation.
(4) The fourth pattern of response can primarily be seen in clinical populations (38). In contrast to redistribution processes, where involved anatomical structures remain the same, reorganization processes include decreased activation in some areas and additional recruitment of new cortical regions. This shift of activation is thought to reflect a process shift: due to training, other cognitive processes become involved in solving the task. Additional recruitment of temporal regions outside the usual WM network in our second responder might reflect such a reorganization process. At the behavioral level, this participant showed higher processing speed (Alertness and SDMT) and visual WM performance potentially resulting from a reorganization process. The increase of activation was more apparent in the left hemisphere. We assume that the individual has developed a verbal coping strategy, which triggered the observed change in activation after training.

**Table 3:** **Summary table of changes from baseline to post-treatment for all participants**.

Case	Change in activation	Processing speed measures		Processing speed and WM (SDMT)	WM measures	
		Tonic alertness (TAP alertness A)	Phasic alertness (TAP alertness B)		Visual WM (corsi blocks bw)	Verbal WM (digit span bw)
TG1		↑	↑			
TG2	↓	↑	↑	↑		
TG3					↑	
TG4		↑	↑	↑		
TG5				↑	↑	↑
TG6	↑	↑	↑	↑	↑	
CG1		↓				↑
CG2		↑	↑		↑	
CG3		↓				↑
CG4		↓	↑			

*empty spaces, no change; ↑, increase; ↓, decrease; bw, backward; TG, training group; CG, control group; WM, working memory*.

It should be noted, that non-responding participants and participants of the CG also showed changes regarding cognitive performance. Changes in the CG might reflect normal variations in performance, since improvement was isolated on single tests and never consistent across all tests within a single cognitive domain. Changes in non-responding participants of the TG in contrast were more systematic. One of these participants showed increased processing speed (Alertness and SDMT), whereas another participant performed better in all WM measures (SDMT and visual and verbal WM). However, these behavioral changes were not accompanied by changes in brain activation and thus triangulation criteria were not fulfilled.

We are aware that this case-series has several limitations. A first limitation is certainly the small sample size. Second, we did not predefine cut-off values for behavioral and fMRI changes. Third, observed changes in brain activation and cognitive performance might be the result of factors not assessed in the study. To exclude at least variations resulting from the circadian cycle, cognitive and fMRI assessment were always performed at the same daytime. Fourth, changes in fMRI might be caused by variability in the method itself (42). That is why these well-known intersession differences were partly controlled by the applied triangulation approach. Fifth, we used a passive CT instead of implementing a shamed training group (TG). Therefore, it cannot be excluded that changes in the two participants in the TG result from multiple factors such as motivation, social interaction, and emotional support. Sixth, only few participants showed significant cognitive deficits when compared to normative data. Thus, higher baseline performance might reduce the potential to observe significant changes induced by cognitive training due to a simple ceiling effect. A last limitation of the present study is that performance data from the *n*-back task during fMRI was missing due to technical problems. Therefore, fMRI activation patterns could not be compared directly to WM and speed performance inside the scanner but only to the performance outside the scanner in terms of a transfer effect.

These limitations might have modified the outcome of our case-series. Still, we were able to identify two different types of changes after cognitive training in patients with early RRMS: (A) a decreased brain activation, which was associated with increased processing speed and (B) a reorganization process, associated with higher processing speed and WM. The occurrence of different or even opposed patterns of response after the same training indicates a problem with traditionally applied group statistics. Different and especially opposed patterns within the same sample will distort results of classical statistical comparisons. Underlying processes may therefore remain concealed.

## Conflict of Interest Statement

This study was supported by a research grant from Bayer Schering AG Switzerland. The funder had no role in the design and conduct of the study; collection, management, analysis, and interpretation of the data; preparation, review, or approval of the manuscript; and decision to submit the manuscript for publication. Ludwig Kappos’s Institution (University Hospital Basel) received in the last 3 years and used exclusively for research support: steering committee, advisory board, and consultancy fees (Actelion, Addex, Bayer Health Care, Biogen, Biotica, Genzyme, Lilly, Merck, Mitsubishi, Novartis, Ono Pharma, Pfizer, Receptos, Sanofi-Aventis, Santhera, Siemens, Teva, UCB, Xenoport); speaker fees (Bayer Health Care, Biogen, Merck, Novartis, Sanofi-Aventis, Teva); support of educational activities (Bayer Health Care, Biogen, CSL Behring, Genzyme, Merck, Novartis, Sanofi, Teva); royalties (Neurostatus Systems GmbH); grants (Bayer Health Care, Biogen, Merck, Novartis, Roche, Swiss MS Society, the Swiss National Research Foundation, the European Union, Roche Research Foundations). Iris-Katharina Penner received honoraria, research, or travel support from Bayer Pharma AG, Bayer AG Switzerland, Biogen Idec, Genzyme, Merck Serono, Novartis, the Swiss Multiple Sclerosis Society, and Teva. Martina Hubacher, Katrin Weier, Markus Stöcklin and Klaus Opwis have nothing to disclose.
